# A load-measuring device can achieve fine-tuning of mediolateral load at knee arthroplasty but may lead to a more lax knee state

**DOI:** 10.1007/s00167-018-5164-3

**Published:** 2018-10-04

**Authors:** William A. Manning, Alasdair Blain, Lee Longstaff, David J. Deehan

**Affiliations:** 1 Newcastle Surgical Training Centre, Freeman Hospital, High Heaton, Newcastle upon Tyne, NE7 7DN UK; 2 Mitochondrial Research Group, Institute of Neuroscience, Medical School, Newcastle upon Tyne, NE2 4HH UK; 3 University Hospital of North Durham England, Durham, DH1 5TW UK

**Keywords:** Sensor technology, Tensiometer, Load, Balance, Knee performance

## Abstract

**Purpose:**

A balanced knee arthroplasty should optimise survivorship and performance. Equilibration of medial and lateral femorotibial load requires guided judicious pericapsular ligament release. The null hypothesis was that there would be no difference between use of a tensiometer device and a remote load sensor final load transfer across the joint through functional arc of motion.

**Methods:**

A cadaveric study, using eight knees, was performed to define the impact of an established gap distraction device against load sensor-aimed soft tissue release in a TKA setting. Using validated measures of laxity in six degrees of freedom and true real-time load sensing four states were examined: native knee, TKA using spacer blocks (TKA), TKA with soft tissue release aided by a monogram tensiometer (TKA-T) and finally where load across the tibiofemoral articulation remains unbalanced final soft tissue release using a sensor device (TKA-OS).

**Results:**

The laxity pattern was equivalent for TKA-T and TKA-OS. However, in only four of these seven knees despite the tensiometer confirming equivalence of rectangular flexion–extension gap dimensions and centralisation of collateral ligament distraction, there remained a > 15lb medial to lateral load difference for at least one point of the flexion arc. This was corrected by further final soft tissue release guided by the OS sensor device in the final three knees.

**Conclusion:**

Tensiometer-guided soft tissue release at two points of flexion failed to achieve balance for three out of seven knee arthroplasty procedures. Sensor technology guided final soft tissue balancing to equilibrate load across the joint through full arc of motion. This work argues for the role of continuous sensor readings to guide the soft tissue balancing during total knee arthroplasty.

## Introduction

Total knee arthroplasty (TKA) remains the treatment of choice for end-stage osteoarthrosis and increasing numbers are being performed [20] with the express purpose of alleviating pain and restoring function [2]. There remains a high incidence of dissatisfaction and this may often be due to poor kinematic performance of the replaced knee due to a lack of balance with excess load across one particular compartment leading to instability and/or pain necessitating early revision [5, 8, 23]. Optimal performance of the replaced knee is reliant upon the complex process of correct positioning of the implants, restitution of osteoarthritic pre-operative deformity and matching of the inherent constraint of the device with the host soft tissue envelope [4, 10, 31]. The final common pathway is correct soft tissue tension. Poor kinematic performance leading to loss of functional control expressed as instability, avoidance of key tasks such as stair climbing and reduced functional activity [24]. During surgery, the active muscle envelope is relaxed and judgement of laxity is reliant predominantly on the passive inherent ligamentous constraint [33]. The surgeon is, therefore, extrapolating ultimate performance from a relaxed non-loaded paralysed state to subsequent loaded weight-bearing performance [11, 32]. To guide such during surgery key measures are taken. These include gap balancing with the use of spacers, a tensiometer distracting the medial and lateral structures and most recently remote sensing of load instrumentation.

Simple reliance upon gap balancing or measured resection or a combination of the two provides only a partial projection of the final performance of the knee [19]. There remains debate about the role of frontal plane single-stance optimal biomechanical alignment in achieving perfect balance [23, 35]. Optimal performance through a full arc of motion is dependent upon consistent load distribution across the mediolateral articulation [17, 21]. Any direct correlation between equivalence of gap balancing, especially when as is standard only performed at two distinct points of motion remains unproven [22, 33]. Newer technology does allow for analysis in real time of load across the two tibiofemoral compartments and this work both in vivo and in vitro must be related to accepted standards of surgical resection and implant positioning [13, 15].

Therefore, a cadaveric study was performed to examine the effect of balancing after measured resection, further guidance from the use of a tensiometer and a remote sensor of load at knee arthroplasty. Such optimisation of load could be considered the mainstay of balance and offers the potential for optimal knee performance following arthroplasty. The null hypothesis was that there would be no difference between use of a tensiometer or a remote sensor device in achieving final load difference(s) between medial and lateral compartments after completion of surgery through a functional arc of motion.

## Materials and methods

A trial of two limbs was used for testing of the system but the results were not included in the final analysis. Eight left-sided lower limbs were obtained from a tissue bank (Science Care Phoenix Arizona 85207). Ethical approval was obtained (Human Tissue Act 2004, Sect. 16/2, license number 12148). The median BMI of the donors was 26, range 18–31, all were male, 6 white Caucasian, 1 African-American and 1 for whom race was not recorded, median age was 65 years, range 57–81. At time of surgery, there was no limb malalignment or malrotation. All limbs exhibited full passive movement and there was no mediolateral, anteroposterior or varus/valgus pattern worse than grade A (< 3 mm) to manual stress testing. No limb had evidence of a scar. Documentation from Science Care stated that there was no record of previous surgery on the index knee. Pre-TKA radiographs were undertaken to exclude degenerative change, peri-articular deformity, exclude the presence of previously implanted metalwork or osseous deformity and to confirm normal anatomy. Direct inspection at arthrotomy confirmed normal chondral appearances.

### Experiment setup

This has been previously reported [11, 17, 21]. Loading of the individual extensor, flexor, adductor muscle groups and lateral constraints was as per previously accepted safe loads in an open-chain fashion with weight over a more proximal pulley system to mimic the hip joint. Navigation trackers were fixed to the femur and tibia at least 10 cm away from the area of resection and in such a position as to not interfere with the surgical field or disrupt visualization sensors nor impinge on the muscle line of pull.

### Stages of experimentation (Fig. 1a)

Laxity assessment was performed for the native knee at 0, 30, 60, 90 and 110 degrees of flexion (native knee). The native knee without arthrotomy was assessed under loading conditions for maximal laxity at each point of flexion for varus/valgus, maximal rotation, total anteroposterior movement. This was as previously reported using maximal manual deformation whilst confirming the laxity pattern for the six degrees of motion at the key measurement flexion points [11]. All limbs exhibited passive flexion from full extension to at least 130 degrees of flexion. A single-radius cruciate retaining Total Knee Arthroplasty (CR TKA) (Stryker Triathlon, Michigan USA) was performed via a medial parapatellar approach using a measured resection technique. The technique has been described previously [hunt manning]. The transepicondylar axis and central trochlear sulcus were used for femoral component rotational referencing. The tibial rotation for all cases was neutral with respect to a line drawn between the medial aspect of the patellar tendon and the PCL footprint [29]. All tibial trays were confirmed to exhibit no lift off and the femoral and tibial components were secured to the cancellous bone through the use of multiple countersunk lag screws after multiple cycles of full motion. The capsulotomy was closed prior to load measurement with either a normal spacer or the Verasense™ spacer of the appropriate depth. The joint line was confirmed as being at the level of meniscal resection for the extended and flexed knee at 0 and 90 degrees of flexion. In all cases patellar tracking was normal without clunk, or maltracking and normal glide after closure with full movement prior to the commencement of any laxity or load measurements.

**Fig. 1:**
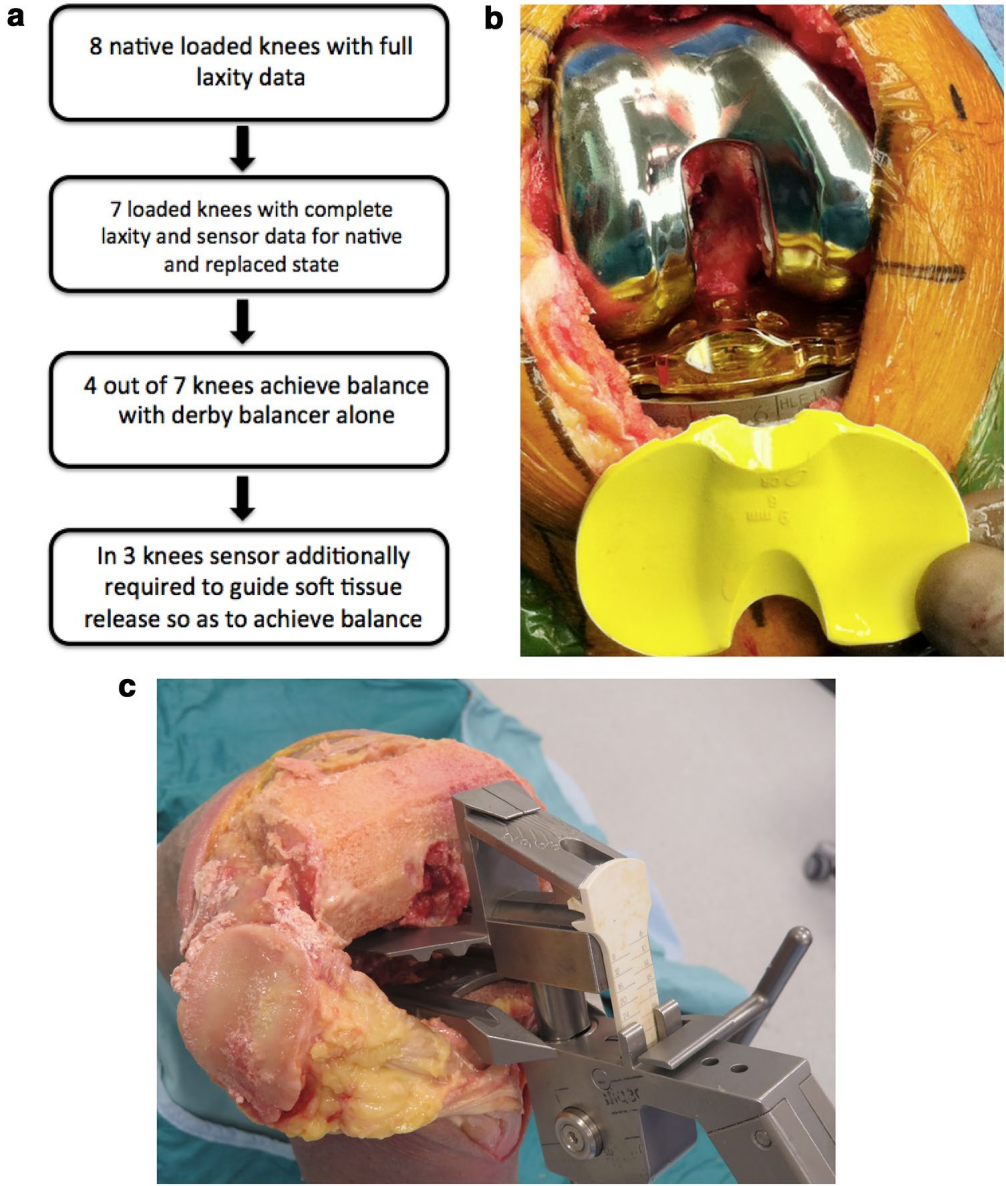
**a** Schematic outline of the study design. **b** Verasense in situ interchangeable with equivalent poly-trial. **c** Views of the tensiometer which defines deviation of load from midline and quantifies magnitude of mediolateral space

A sensor Verasense™ (Orthosensor, Dania FL) of appropriate thickness was activated and replaced the polyethylene trial on the tibial tray (Fig. 1b). For this and subsequent states, a control medial and lateral tibiofemoral contact forces were recorded as the loaded knee was taken through a range of passive flexion and this was repeated for all states at least on three occasions for all states [11]. No soft tissue release was required to achieve the criteria outlined above and capsular closure was performed. The median of three measurements was taken as the median. The use/calibration and limitations of the Verasense™ have previously been reported in both clinical and cadaveric loaded setting [15, 23].

The capsule was then opened, the femoral and tibial components were removed and the monogram tensiometer (Fig. 1c) used to define full extension and flexion at 90° rectangular space as previously described [1]. Following this, symmetrical tension is applied to the joint line in extension using the ligament tensor [9]. This symmetrical tensioning was able to identify any residual varus/valgus deformity and could determine the magnitude of the gap. Through a process of further femoral resection and new chamfer cuts [*n* = 1] (tight extension and loose flexion) or medial soft tissue release (elevation of the posterior component of the deep aspect of the medial collateral ligament) the replaced knee was considered balanced as per the tensiometer (TKA-T) [hunt]. Each cadaveric knee was repeatedly measured until the flexion and extension gaps were equal and the pointer faced centrally and north in both states. This was taken as a tensiometer-balanced knee (TKA-T). This was achieved for all seven knees. The components were again reinserted, secured to the cut bone and an appropriate polyethylene insert trialled to confirm the minimal criteria as set previously above. Again the capsule was closed with running heavy PDS and the same laxity and load measurements were performed as before. Each measurement for each point of knee motion was taken three times and the load-measuring device ‘zero-d’ between each measurement. It was essential for reliability of measurement to avoid overloading or loading the device beyond 90lbs at any point. If during the measurement of load there was at any point of flexion 0, 30, 60, 90 or 110 a side to side difference of > 15lbs this knee was classed as not balanced as per the Verasense™ and these results were removed from this subgroup analysis. For four of the seven knees, the use of the tensiometer allowed for a balanced knee [15, 32].

For the remaining ‘unbalanced’ (defined as one or more points of measurement where there was a > 15lbs mediolateral compartmental load difference) knees (*n* = 3), further soft tissue-only release was performed through periosteal longitudinal elevation/release of the posterior fibres of the superficial medial collateral ligament [16, 17]. This was carried out after the capsule had been opened but the components were left in situ and repeat load measurements were made with capsule closure until a true load balanced state was achieved. This state where final soft tissue release, as guided by a sensor, was required to ensure no side to side difference in load of > 15lbs was taken as a sensor balanced knee (TKA-OS). At this point for each knee repeat complete laxity and load measurements as before were performed.

The senior authors (LL/DJD) performed all surgical procedures and stress testing. Computer navigation was a Stryker eNdtrac Knee Navigation System, Michigan, USA, allowing for tracked knee motion to an accuracy of ± 0.5 mm. The average time between two digitizations was approximately 150 ms, which equated to a frame rate of 6.67 Hz m [6]. The Verasense™ tibial sensor recorded tibiofemoral contact force (lbs/force) and contact points (mm accuracy ± 2 mm—C.Anderson, OrthoSensor). This was taken for 0, 30, 60, 90 and 110 degrees of flexion.

Knees were manually stressed to mimic intraoperative laxity assessment, with the endpoints defined as the maximal displacement achieved [11, 15, 17]. The primary TKA in extension acted as the datum from which maximal displacements of the tibia in relation to the fixed femur were recorded in six degrees of freedom [14]. For each knee condition maximal manual varus, valgus, internal and external (IRER) displacements were each recorded at five angles of flexion (0°, 30°, 60°, 90° and 110°). Repeated flexion/extension cycles were undertaken between measurements to reduce hysteresis and ensure compartment forces remained constant during passive flexion [11, 32].

### Statistical analysis

Four distinct states were examined: native knee (laxity patterns), primary knee arthroplasty (laxity and load data)—TKA; primary knee arthroplasty with soft tissue release to achieve balance and equal tibiofemoral load—TKA + T; primary knee arthroplasty with failed balancing using tensiometer and further adjustment made using tibiofemoral load sensor—TKA + OS. Measurements were taken at 0, 30, 60, 90 and 110 degrees of flexion. Data processing and analysis were performed using R statistical software [28]. A linear modelling approach and Student’s *t* tests were used to compare differences in tibiofemoral force and contact point measurements [25]. Significance was set at a level of *p* < 0.05. Power analyses from previous studies have determined eight limbs sufficient to identify significant differences with 95% confidence and 80% power [11, 18].

## Results

For the purposes of defining the key states during surgery, a ‘balanced’ knee was defined as one where there was never a greater than 15lb load difference between the medial and lateral compartments at any point of the flexion arc.

### Laxity findings

#### Anterior drawer laxity

**Fig. 2:**
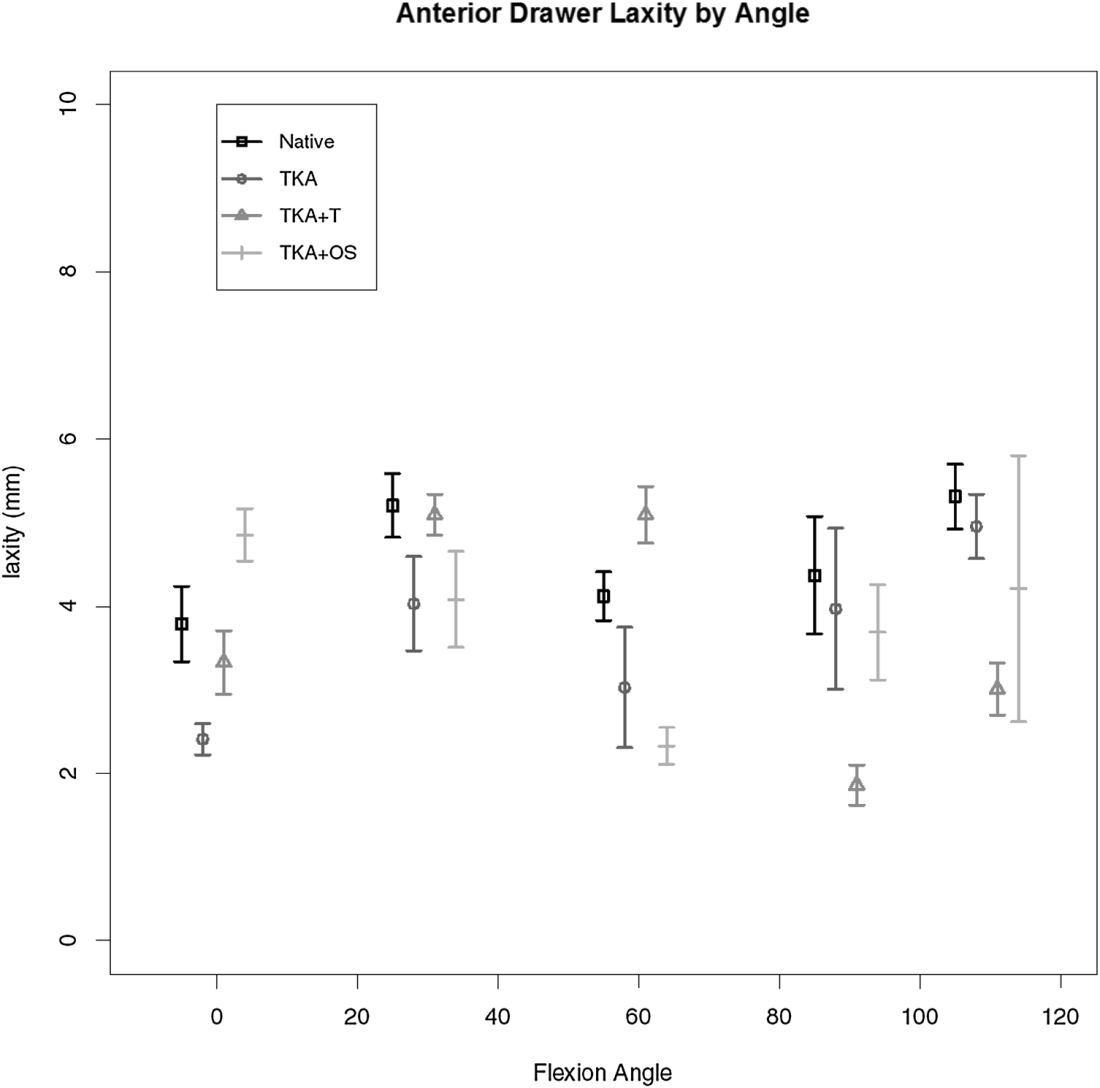
Maximal anterior drawer laxity for each state through range of motion

This is given in Fig. 2 for all four states. When assessed at 90 degrees of flexion, no significant difference at any point of flexion measured.

#### External and internal rotational laxities

**Fig. 3:**
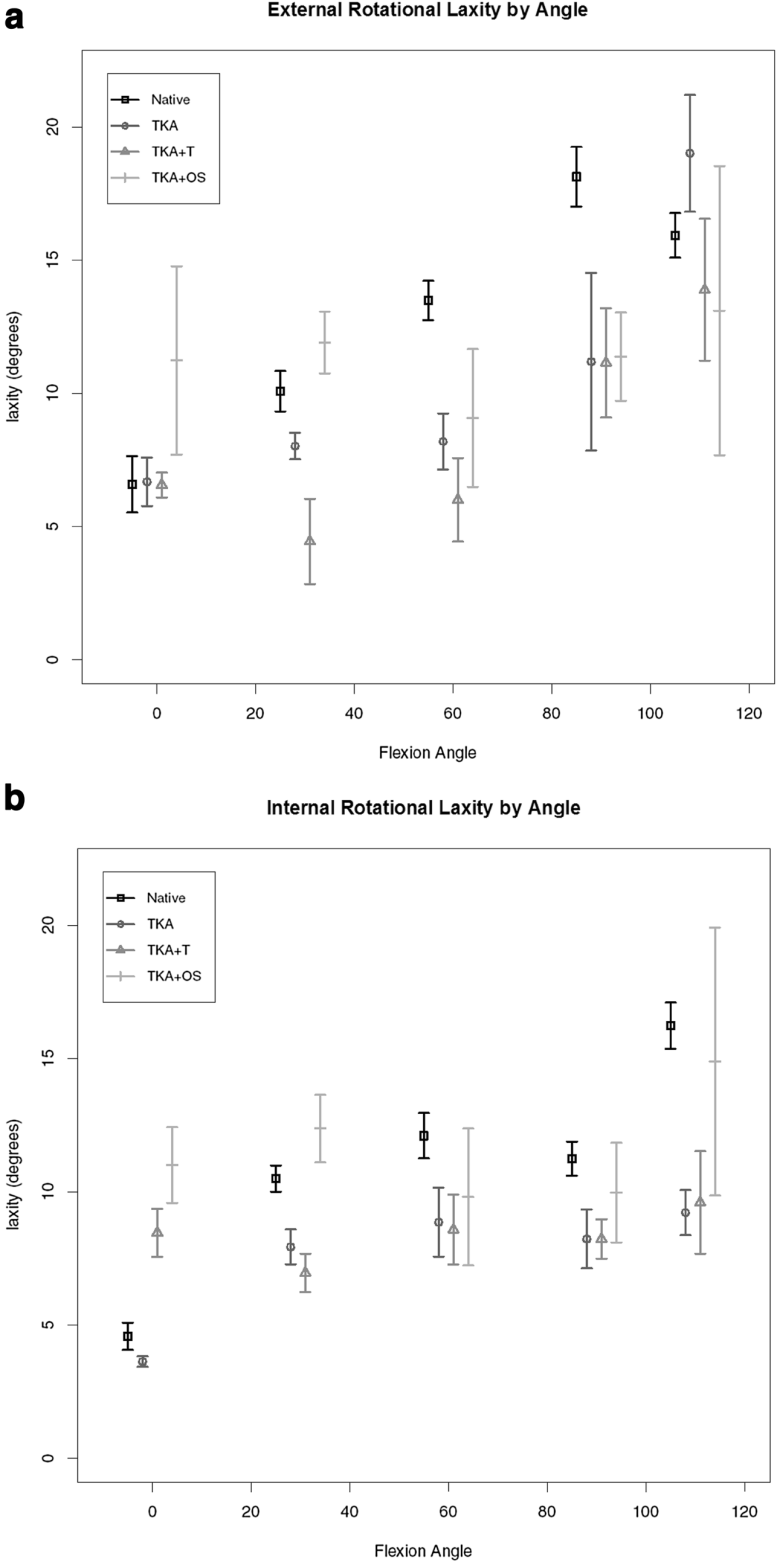
**a** Maximal external rotational laxity by flexion angle. **b** Maximal internal rotational laxity by flexion angle

Maximal external rotation (ER) findings for each point of flexion are given in Fig. 3a. A consistent pattern of increased laxity in early flexion was found for the knee arthroplasty unbalanced state compared to native knee but this was not altered with the use of the tensiometer but only in early flexion 0–30 arc there was a significant increase in laxity for external rotation after further release performed with the guidance of the Versense™ device. Maximal internal rotation results (IR) are shown in Fig. 3b. The only difference for laxity pattern was found at the 0–30 arc for those final knees which underwent middle third superficial MCL release. The native knee exhibited the greatest range of laxity consistent with biological variation. A very similar pattern of maximal laxity was found for the replaced knee and tensiometer-guided knee arthroplasty. Beyond 30 degrees of flexion no significant was found.

#### Valgus and varus maximal laxities

**Fig. 4:**
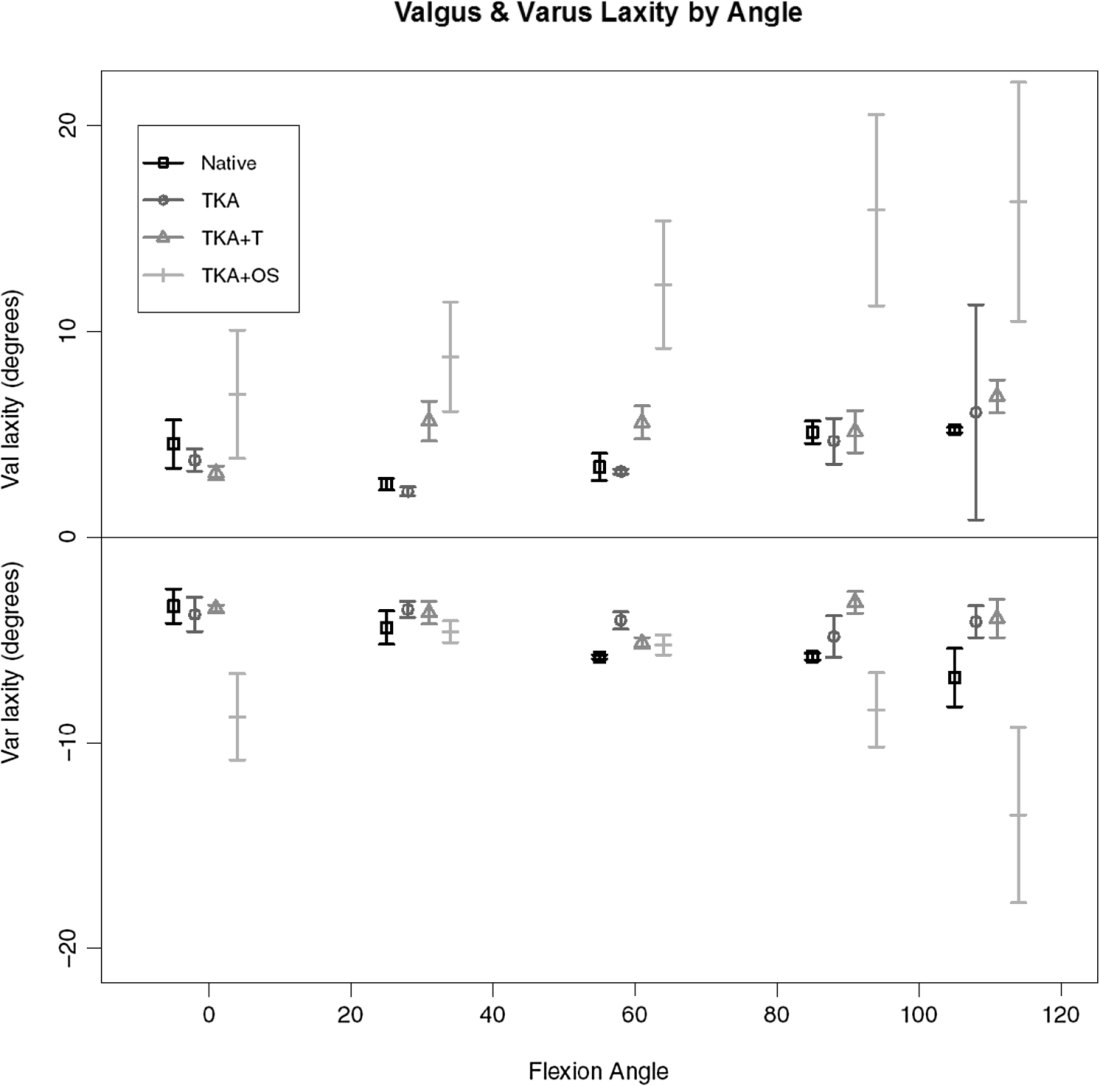
Varus and valgus deformational laxities

Valgus and varus laxity patterns are given in Fig. 4. There was a consistently greater amount of laxity for valgus deformation in the Versense™-guided knee compared to all other. At full extension and 110 degrees of flexion, the Versense™-guided knee arthroplasty exhibited a significantly greater varus laxity compared to the other three states but in the mid-range there was no difference.

### Tibiofemoral load findings

#### TKA + T

**Fig. 5:**
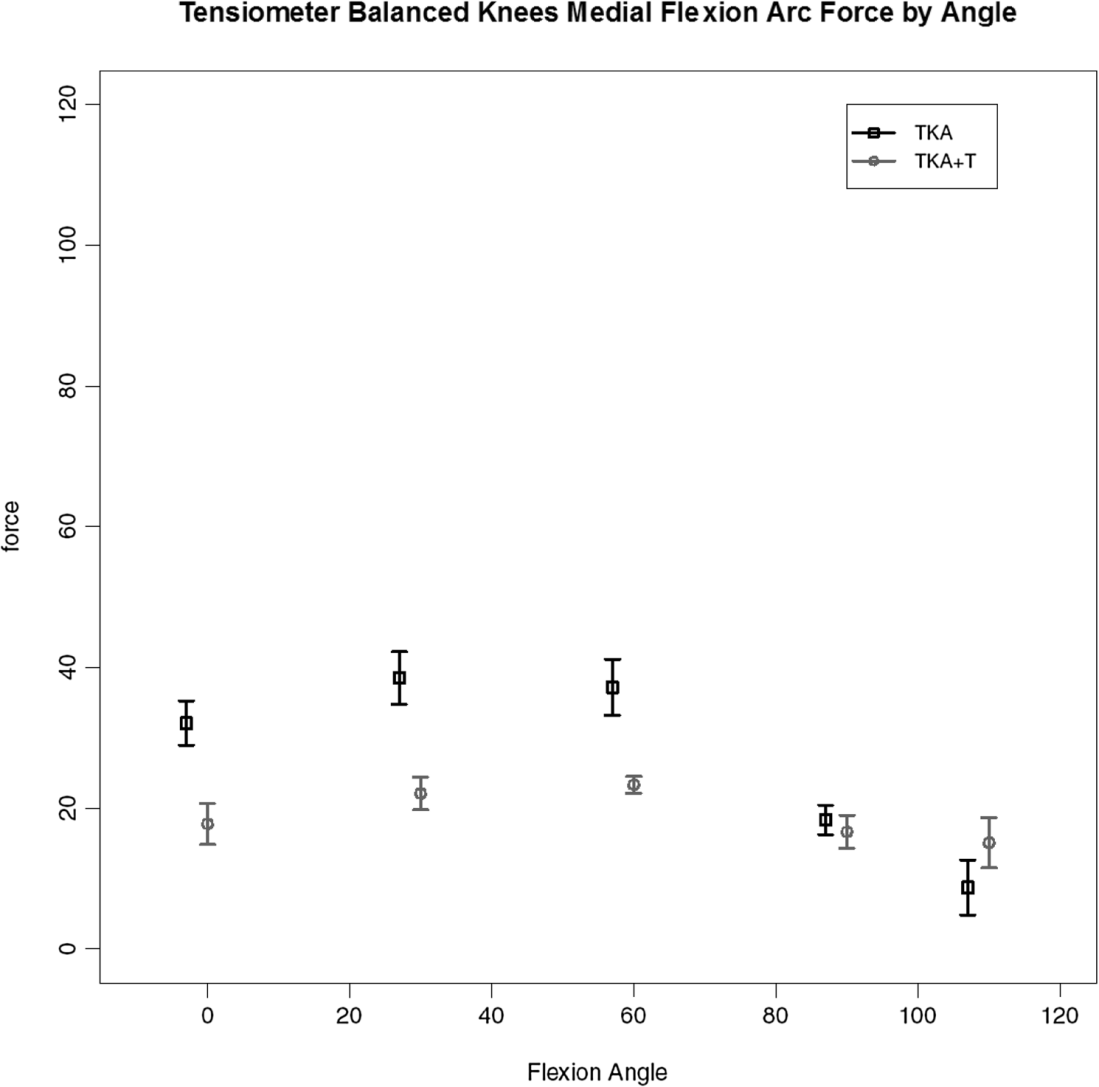
Load across the medial compartment for those knees (TKA-T) balanced with use of tensiometer alone

**Fig. 6:**
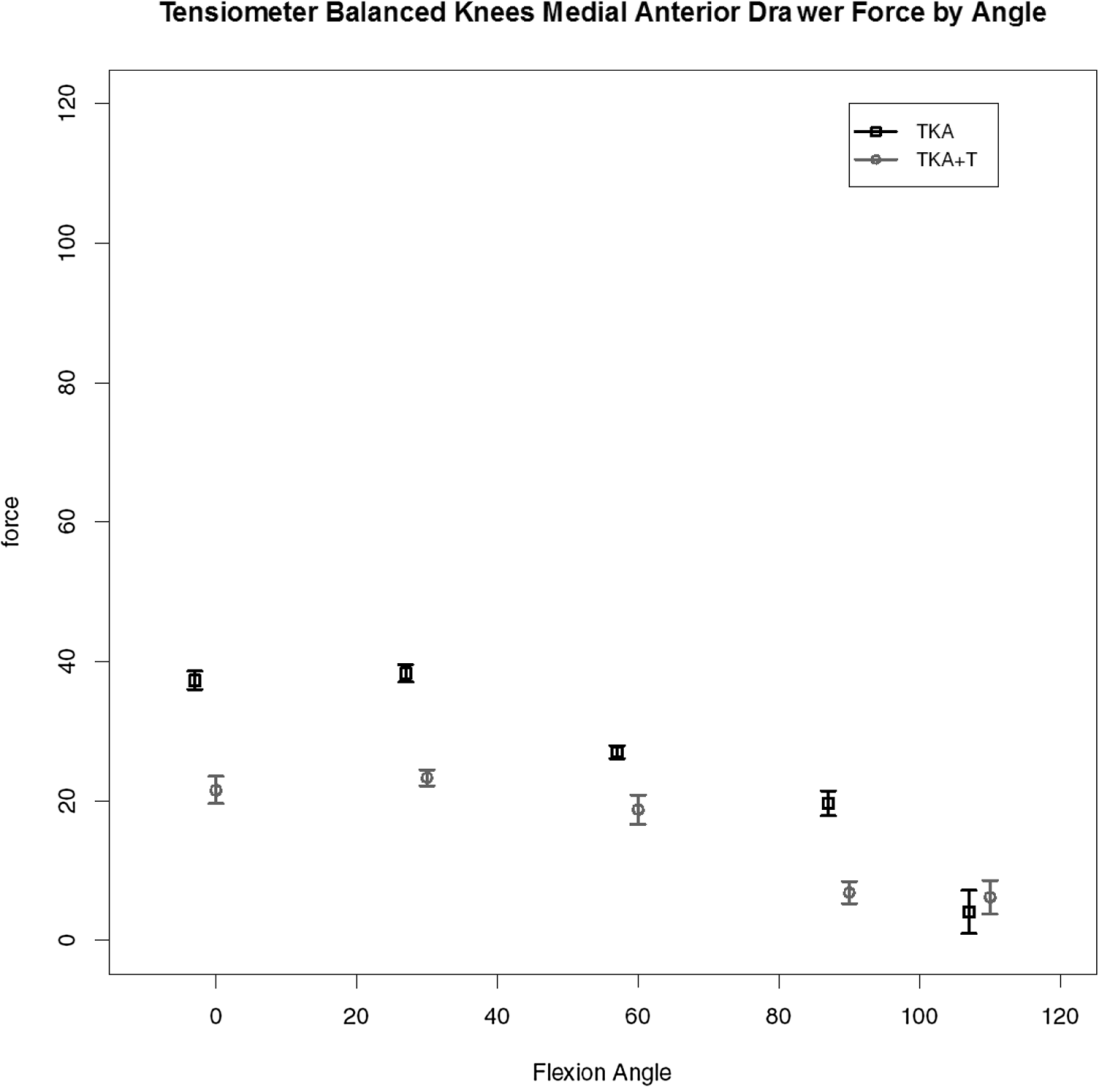
Medial compartment load by flexion angle for TKA-T group under maximal anterior drawer stress

**Fig. 7:**
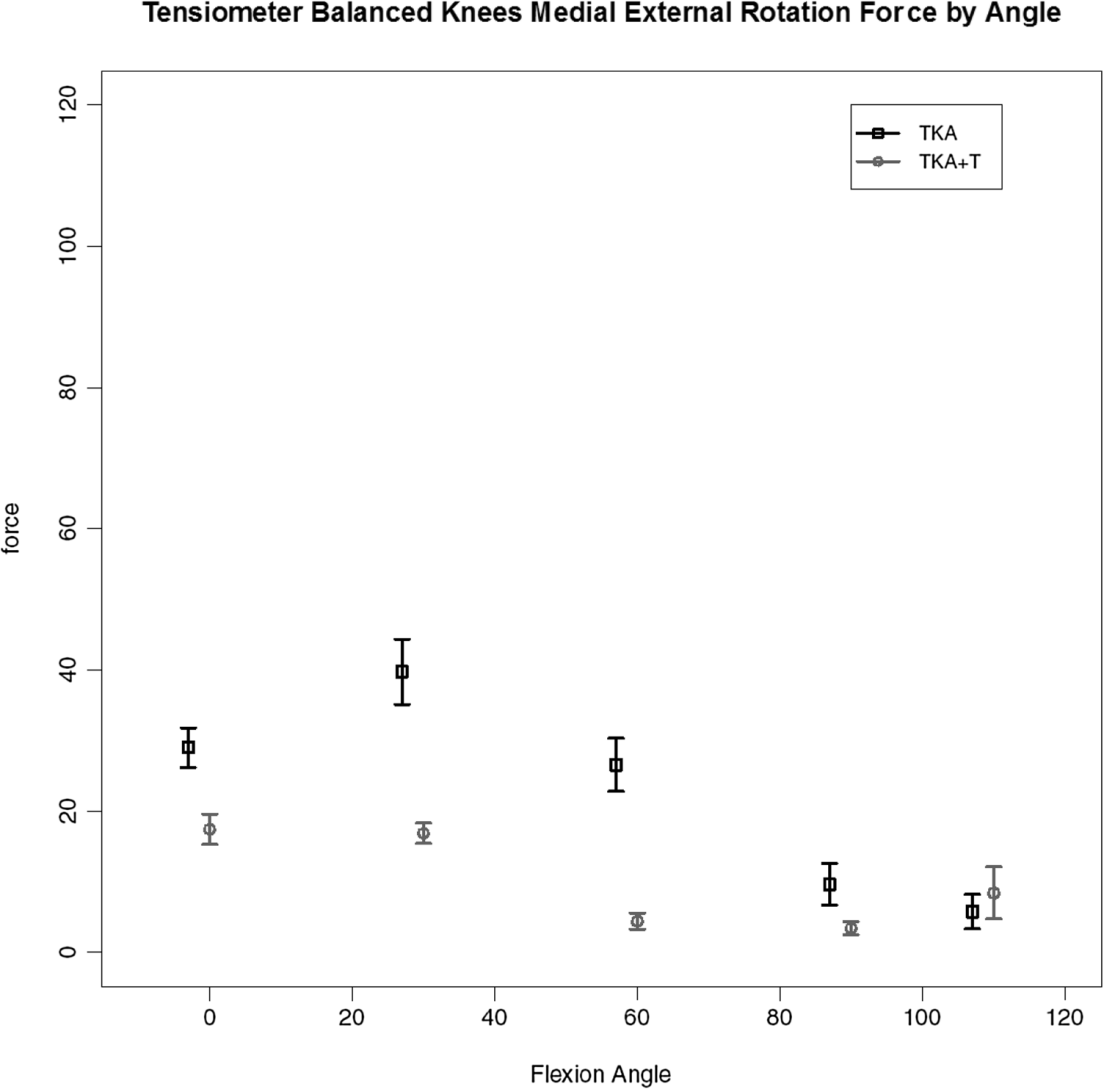
Medial compartment load by flexion angle for TKA-T group under maximal external rotational deformation

For the four knees (out of the seven completed with validated data) where the use of tensiometer alone achieved a balanced knee (< 15lbs load difference between medial and lateral compartments for all measured points) the load data are given. A consistent pattern of ‘smoothing out’ of the curve and reduction in load was found with use of the tensiometer as represented by Fig. 5, medial load (medial flexion arc force by angle); Fig. 6, medial anterior drawer force (load across the medial compartmental under maximal anterior drawer deformation); Fig. 7, medial compartment load with external rotation force. These key graphs confirmed that in four of the seven knees after soft tissue release (deep medial capsular elevation from the superior edge of the medial tibia) use of the tensiometer was sufficient to enable a balanced state. These graphs exhibited very similar shape changes.

#### TKA + OS

**Fig. 8:**
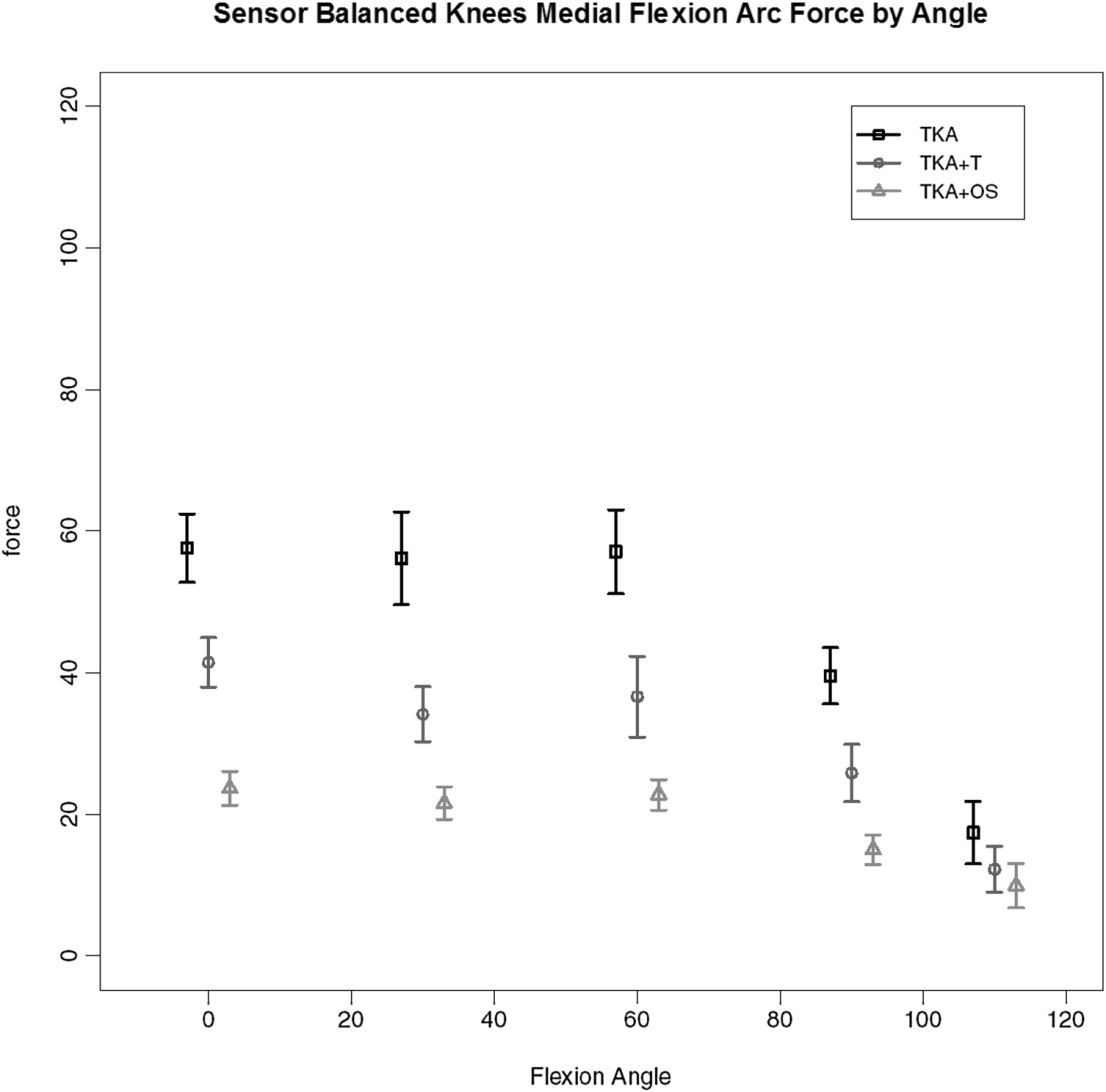
Medial compartment load by flexion angle for those knees (TKA-OS) requiring Verasense™ to achieve balance

**Fig. 9:**
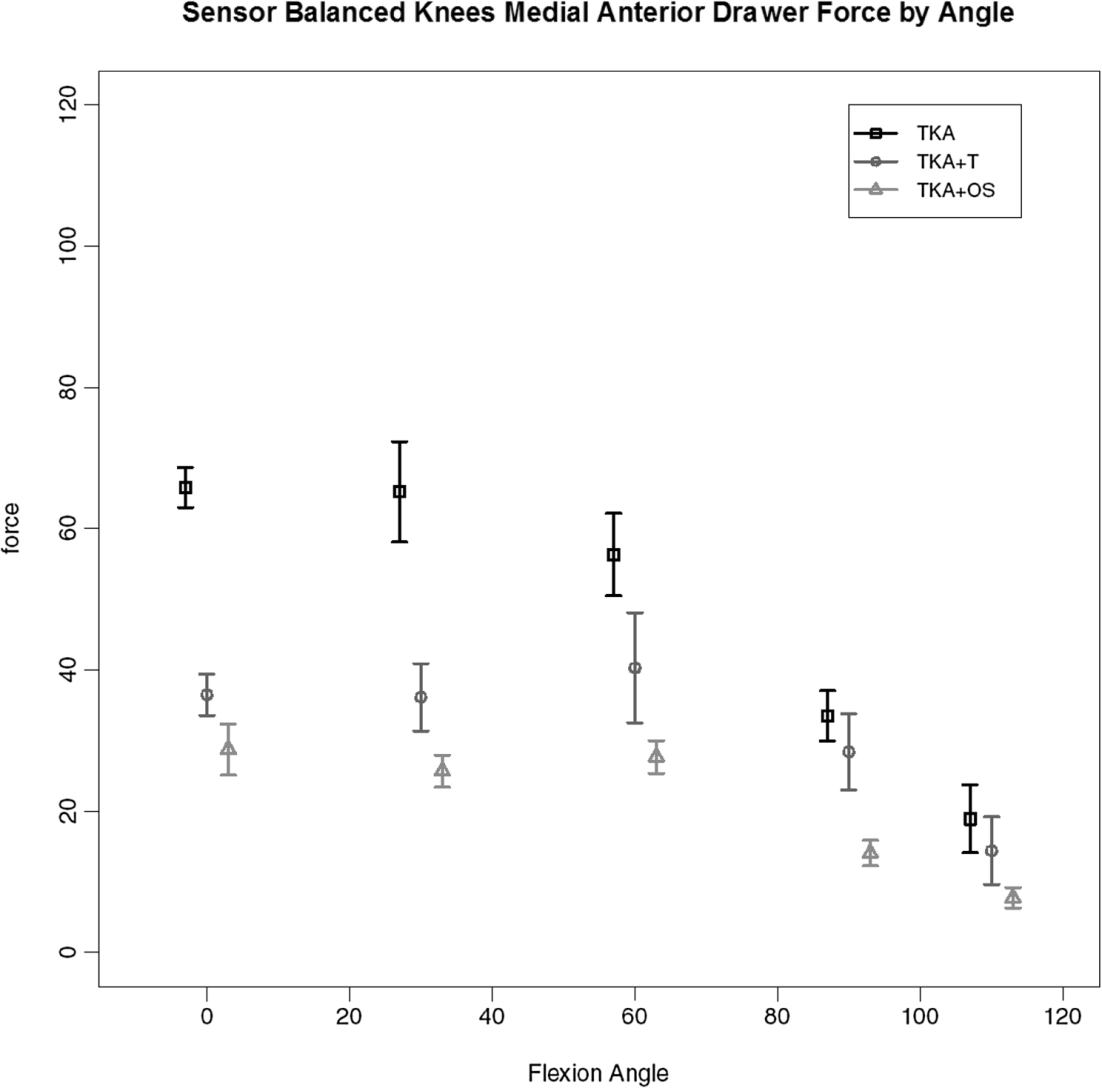
Medial compartment load by flexion angle for those knees (TKA-OS) under maximal anterior drawer stress

**Fig. 10:**
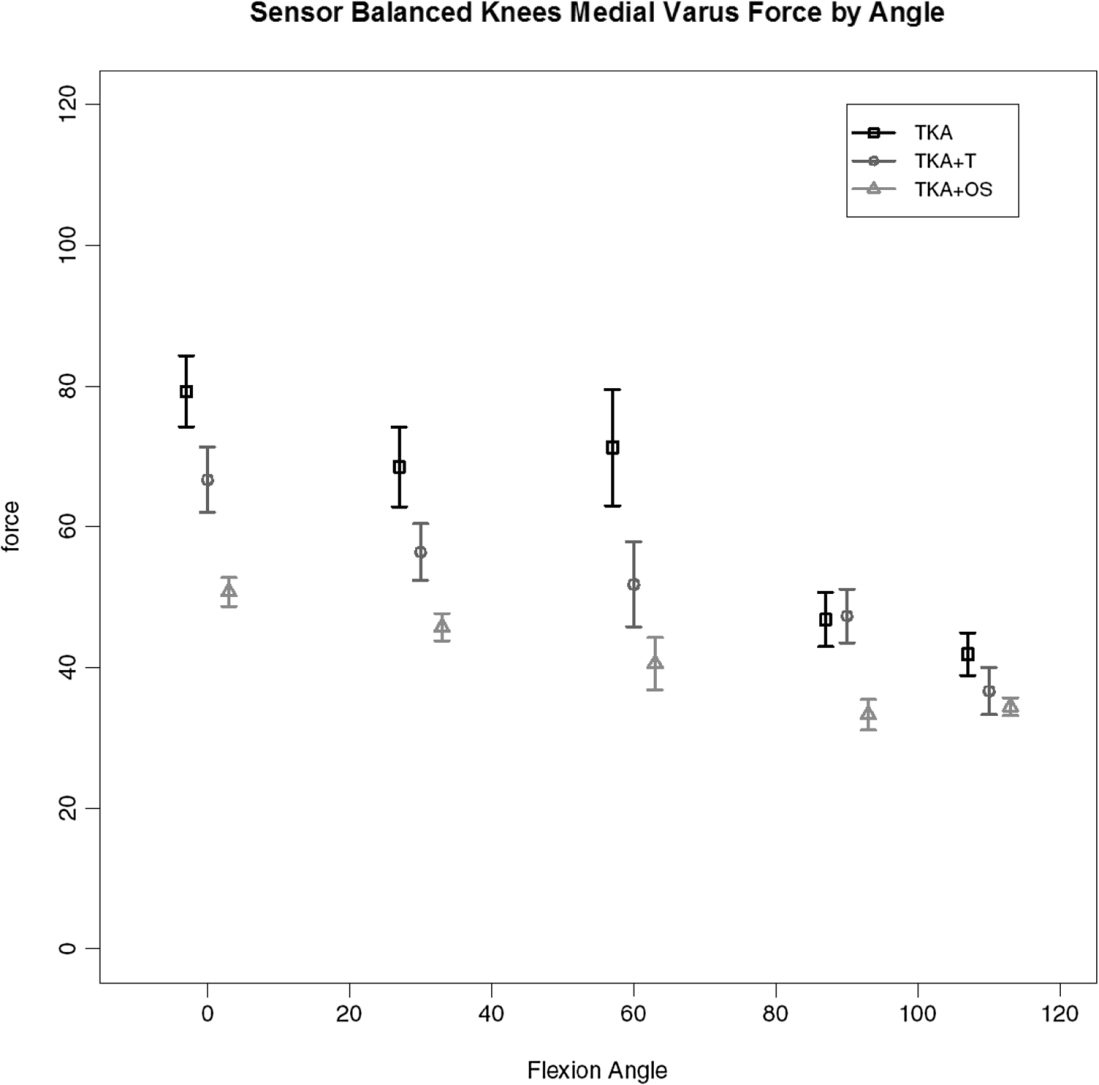
Medial compartment load by flexion angle for TKA-OS group with varus deformation

Of the 7 replaced knees 3 required the additional use of the Verasense™ device to guide further soft tissue release (and in one case additional distal femoral resection) to achieve similar load transfer across the two compartments through full movement. The load across the medial compartment for the key stages of balancing for these three knees is given in Fig. 8. It can be seen that there is gradual reduction in the total load (area under the curve), first with the use of a tensiometer and then with the use of the Verasense™. No such effect was found for the lateral compartment where no significant differences in load under the different states were found. Under deformation through arc of motion the additional effect of further surgical release for these knees so as to achieve mediolateral load equivalence can be seen. A consistent reduction in maximal load across the medial compartment, most evident between 0 and 60 degrees of flexion is found for medial compartment load with anterior drawer (Fig. 9) and medial compartment load for varus loading (Fig. 10).

## Discussion

The principal finding of this work was the absence of a significant change in laxity pattern between the use of a tensiometer and a load-measuring device beyond 30 degrees of flexion. In three out of the seven knee surgical procedures, where complete was available, there was a failure to achieve equivalence of load across the mediolateral articulation through a full arc of motion. With the use of a remote sensor load measuring device any residual mediolateral imbalance was abolished without inducing an adverse laxity pattern.

Previous studies have confirmed the relevance of soft tissue integrity for knee arthroplasty kinematic performance [1, 11, 13, 15, 22, 32]. An imbalanced knee arthroplasty may lead to loss of motion, pain and instability with risk for early revision [3, 26]. The current work argues that modern tensiometers in isolation may fail to reliably equilibrate load across the medial and lateral compartments. Distraction tensiometers may allow the surgeon to achieve rectangularity of flexion and extension gaps but are very easily influenced by rotational error and further only measure soft tissue behaviour at two points of motion [13, 23]. A tensiometer device aims to achieve balance between the medial and lateral compartments through distraction of the soft tissue envelope in a static format. Determination of load continuously as achieved through a remote sensor device both smoothes out the curve and reflects the varying contributions of the collateral ligament subcomponents to resistance to distraction and thereby load transfer. In this work, three out of seven were found to be imbalanced despite achieving equivalence for flexion–extension gap balance at both full extension and 90 degrees of flexion. The single radius design used could be expected to offer predictable kinematics during this arc but this was not found for these test conditions [18, 31]. The side to side difference for this tensiometer subgroup was most marked at 60 degrees of flexion. This device is very sensitive to rotational changes explained by the differing elastic constraints of the three components of the medial collateral ligament and further by the action of the posterolateral corner. Posterior translation of the tibial component may also influence flexion gap dimensions still further limiting the value of single point flexion measurements to assess balance [19]. The tensiometer only gives a two-dimensional assessment and fails to reflect load distribution through a full arc of motion. It is a visual indicator and as such remains imprecise and is not sensitive to forces across the tibiofemoral articulation even when the joint is not loaded through muscle action. Such discrepancy would be expected to be accentuated in the weight bearing or loaded in vivo situation. The equivalence of distraction does not necessarily equate to load. Such a presumption would require equivalent Young’s moduli for the medial and lateral collateral ligamentous structures [4, 30]. However, previous work has found conflicting values for such. It is postulated that this may be the additional influence of the posterolateral corner on the lateral collateral ligament and the differing lines of action of the components of the MCL [30, 34].

There are key limitations to the interpretation of this work. No knee specimen exhibited pre-existent chondral loss or deformity which may not necessarily reflect the true clinical scenario. All cadaveric specimens were male and gender may influence findings from cadaveric work [7]. Whilst the custom-made jigs did ensure that the line of pull of the muscle group accurately replicated that found in vivo, subphysiological loads were used, as in previously published works, so as to minimise the risk of muscle disruption [17]. Time zero studies fail to reflect soft tissue elastic changes which occur with adaptive response to load over time.

The key arc of discrepancy between the two measurement systems was at 30°–60°. Previous work has found that there is a unique pattern of biomechanical behaviour at the tibiofemoral articulation between 30 and 90 degrees of flexion [27]. Recent work increasingly supports the view that continuous measurement through full arc irrespective of femoral or polyethylene insert geometry is of value [22]. Indeed, normal walking pattern is performed predominantly during the swing phase of 60°–80° and stair climbing requires stability of the knee for anteroposterior movement at 80° [12]. Any mediolateral imbalance is likely to lead to poor performance and avoidance of such tasks.

## Conclusion

This work supports the view that conventional two-point-of-motion flexion/extension gap balancing is inadequate. The remote sensor Verasense™ device specifically improved load distribution in the arc of motion and as such may be considered an essential component of the surgical instrumentation at total knee arthroplasty.
